# Angiogenic response in an *in vitro* model of dog microvascular endothelial cells stimulated with antigenic extracts from *Dirofilaria immitis* adult worms

**DOI:** 10.1186/s13071-019-3570-0

**Published:** 2019-06-24

**Authors:** Tatiana Zueva, Rodrigo Morchón, Elena Carretón, José Alberto Montoya-Alonso, Alexis Santana, María Dolores Bargues, Santiago Mas-Coma, Alicia Rodríguez-Barbero, Fernando Simón

**Affiliations:** 1 Group of Animal and Human Dirofilariosis, Parasitology Area, Faculty of Pharmacy, University of Salamanca, Salamanca, Spain; 2 Faculty of Veterinary Medicine, Research Institute of Biomedical and Health Sciences (IUIBS), University of Las Palmas de Gran Canaria, Las Palmas de Gran Canaria, Spain; 3 Albea Veterinarios, Carretera a las Torres, 19, Las Palmas de Gran Canaria, Las Palmas Spain; 4 Department of Parasitology, Faculty of Pharmacy, University of Valencia, Valencia, Spain; 5 Department of Physiology and Pharmacology, Biomedical Research Institute of Salamanca (IBSAL), University of Salamanca, Salamanca, Spain

**Keywords:** Angiogenic factors, Pseudocapillaries formation, *Dirofilaria immitis* antigenic extracts, *Wolbachia* amount, Canine microvascular endothelial cells

## Abstract

**Background:**

Angiogenesis can occur under pathological conditions when stimuli such as inflammation, vascular obstruction or hypoxia exist. These stimuli are present in cardiopulmonary dirofilariosis (*Dirofilaria immitis*). The aim of this study was to analyze the capacity of *D. immitis* antigens to modify the expression of angiogenic factors and trigger the formation of pseudocapillaries (tube-like structures) in an *in vitro* model of endothelial cells.

**Methods:**

The expression of VEGF-A, sFlt, mEndoglin and sEndoglin in cultures of canine microvascular endothelial cells stimulated with extract of adult worms of *D. immitis* obtained from an untreated dog (DiSA) and from a dog treated for 15 days with doxycycline (tDiSA), was determined by using commercial kits. The capacity of pseudocapillary formation was evaluated analyzing cell connections and cell groups in Matrigel cell cultures stimulated with DiSA and tDiSA. In both cases non-stimulated cultures were used as controls.

**Results:**

First, we demonstrated that worms obtained from the dog treated with doxycycline showed a significantly lower amount of *Wolbachia* (less than 60%) than worms removed from the untreated dog. Only DiSA was able to significantly increase the expression of the proangiogenic factor VEGF-A in the endotelial cells cultures. None of the *D. immitis* extracts modified the expression of sFlt. tDiSA extract was able to modify the expression of the endoglins, significantly decreasing the expression of the pro-angiogenic mEndoglin and increasing the anti-angiogenic sEndoglin. The formation of pseudocapillaries was negatively influenced by tDiSA, which reduced the organization and number of cellular connections.

**Conclusions:**

The ability of antigens from adult *D. immitis* worms to modify the expression of pro and anti-angiogenic factors in endotelial cell cultures was demonstrated, as well as the trend to form pseudocapillaries *in vitro*. The capacity of stimulation may be linked to the amount of *Wolbachia* present in the antigenic extracts.

## Background

Angiogenesis consists of the development of new vessels from pre-existing vascular structures, naturally occurring during embryonic growth and in pathological situations, in response to different stimuli including hypoxia, inflammation or tissue injuries [1]. Angiogenesis involves a finely regulated sequence of morphogenetic changes that mainly affect endothelial cells. When this happens, cells produce angiogenic factors which stimulate the onset of angiogenesis in the nearby vessels [2, 3]. During the process both upregulation of proangiogenic factors and downregulation of anti-angiogenic factors occur [3]. One of the main proangiogenic factors is VEGF-A [4] which is one of the five isoforms of VEGF, a homodimeric glycoprotein with mitogenic action of endothelial cells. sFlt-1 is a tyrosine kinase with antiangiogenic capacity [5] related to its ability to decrease the concentration of free VEGF. Endoglins are glycoproteins that are involved in various physiological processes such as cell proliferation, extracellular matrix synthesis, immune response or angiogenesis. The membrane-bound form (m-Endoglin) has a proangiogenic effect [2], while the soluble form (s-Endoglin) shows anti-angiogenic effects [6].

Cardiopulmonary dirofilariosis (heartworm disease) caused by *D. immitis* is a vector-borne transmitted zoonosis that primarily affects canines and felines and is accidentally transmitted to humans [7]. Adult worms lodge in the pulmonary arteries of the infected dogs cause tortuosity, loss of elasticity, proliferative endarteritis and, in addition, pulmonary thromboembolisms when adult worms die. All these anatomical changes at the vascular level leads to luminal obstruction and infarction, decrease of the blood flow, hypoxia, chronic pulmonary edema and hypertension and congestive heart failure [8, 9]. Moreover, the presence and death of microfilariae in renal capillaries is associated with inflammatory processes that damage the renal function [10]. Like other filarial species, *D. immitis* harbors intracellular symbiont bacteria of the genus *Wolbachia* [11] whose contribution to inflammatory processes is key [7, 12]. Since doxycycline reduces *Wolbachia* populations, the administration of this tetracycline is recommended in addition of the adulticide protocol to decrease the inflammatory effects of bacteria released from dying worms [13, 14].

On the other hand, it has been shown that mechanisms developed by *D. immitis* that primarily contribute to their survival and limit damage to the host, can cause long-term deleterious effects on the host [15]. Investigating the regulation of the fibrinolysis system of the hosts by *D. immitis*, it has been demonstrated that the binding of plasminogen by excretory/secretory antigens of the parasite activates the production of plasmin, enzyme responsible for the lysis of fibrin clots. Nevertheless, the long-term production of plasmin increases *in vitro* endothelial cell proliferation, migration and destruction of the extracytoplasmic matrix, all of which are related to vascular remodeling [16, 17].

*Dirofilaria immitis* is able to remodel its vascular habitat in different ways. On the other hand, the pathogenic processes of cardiopulmonary dirofilariosis trigger the appearance of key factors in the stimulation of angiogenesis such as inflammation and hypoxia. Considering all these facts, in this paper we examined the influence of different antigenic extracts of *D. immitis* adult worms on the expression of some pro- and anti-angiogenic factors and their ability to stimulate the formation of pseudocapillaries by using an *in vitro* model of microvascular endothelial cells.

## Methods

### Reagents

Two antigenic extracts from *D. immitis* adult worms were obtained: one from a naturally infected dog (DiSA) and one from a dog naturally infected and treated with doxycycline (10 mg/kg BID for 15 days) (tDiSA). In both cases, worms were removed directly from the right ventricle using the technique of Ishiara et al. [18] with some modifications. Worms were washed, macerated and sonicated (three cycles of 70 kHz, 30 s) in sterile saline solution. The homogenate was centrifuged at 16,000×*g* for 30 min. The supernatant was dialyzed against 0.01 M PBS, pH 7.2. All procedures were carried out at 4 °C.

The presence and concentration of *Wolbachia* in the worms obtained from treated and non-treated dogs were analyzed by real-time PCR as previously described by Simoncini et al. [19]. In brief, first-strand cDNA was generated from 1 µg of total RNA using poly-dT as primers with the M-MLV reverse transcriptase (Promega Biotech Ibérica, Madrid, Spain). Real-time PCR was performed in triplicate. Each 20 µl reaction contained 300 ng of cDNA, 400 nM of each primer and 1× iQ SybrGreen Supermix (Bio-Rad, Hercules, CA, USA). Standard curves were run for each transcript to ensure exponential amplification and to rule out non-specific amplification. Primer sequences were: ftsZ-forw (5′-CGA TGA GAT TAT GGA GCA TAT AAA-3′) and ftsZ-rev (5′-TTG CAA TTA CTG GCG CTG C-3′).

### Cell culture and stimulation of endothelial cells

Canine primary lung microvascular endothelial cells (CPLMEC) from Cell Biologics (Chicago, USA) were grown in Endothelial Cell Medium (Cell Biologics, Chicago, USA) supplemented with Endothelial Cell Medium Supplement Kit (0.5 ml of VEGF, 0.5 ml of EGF, 5.0 ml of L-glutamine), 2% fetal bovine serum (FBS; Cell Biologics), 50 U/ml penicillin and 50 μg/ml streptomycin. Plates were pre-coated with 0.1% pig gelatine (Sigma Chemical Co., San Luis, USA). Cells were cultured at 37 °C in a humidified atmosphere in the presence of 5% CO_2_/95% air. The medium was changed every 3 days. Expansion was carried out by trypsinizing the cells (Trypsin/EDTA, Cell Biologics) and re-plating them when the proliferating cells had reached a sufficient density. Passaging was performed the ratio of 1:3. Cell counts were performed using a Countess® Automated Cell Counter (Invitrogen, California, USA) following the manufacturer’s instructions.

CPLMEC were treated as previously described [20]. In brief, endothelial cells (10^6^ cells/plate) were plated on 100 mm culture plates and were grown for 4 days to obtain confluent cultures and treated with 1 μg/ml of DiSA or tDiSA for 24 h. Non-stimulated cells were used as controls in the same conditions. Subsequently, hypoxia was induced for 24 h, replacing O_2_ in the air with an inert gas in a hypoxia chamber. Finally, the supernatant of the cell cultures was collected and CPLMEC were lysed in ice-cold lysis buffer [20 mM Tris-HCl (pH 7.5); 140 mM NaCl; 10 mM ethylendia-minetetraacetic acid; 10% glycerol; 1% Igepal CA-630; aprotinin, pepstatin, and leupeptin at 1 μg/ml each; 1 mM phenylmethylsulfonyl fluoride and 1 mM sodium orthovanadate].

### Angiogenic factors assays

VEGF-A, sFlt, mEndoglin and sEndoglin concentration in the endothelial cells culture medium were measured by ELISA using a Canine VEGF Quantikine ELISA kit (R&D Systems, Minneapolis, USA), Dog CD105 ELISA kit (LSBio, Seattle, USA), Canine Soluble Fms-Like Tyrosine Kinase Receptor 1 ELISA kit (MyBioSource, San Diego, USA) and Dog CD105 ELISA kit (LSBio), respectively, following the manufacturers’ instructions. The results are presented as the mean ± SEM of three experiments performed in duplicates.

### Endothelial cell tube formation assay

Endothelial cell tube formation was assessed as previously described by Jerkic et al [21]. In brief, a total of 8000 CPLMEC per well were plated on Matrigel® precoated plates (BD Biosciences, San José, California, USA) and cultured in Endothelial Cell Medium supplement. Half an hour later, DiSA or tDiSA was added in the wells (1:10 dilution). After seeding on Matrigel®, cells spread and aligned with each other to develop hollow, tube-like structures. The cells and intercellular junctions were observed each hour for 7 h of incubation and the morphological changes were photographed at 3 h using a phase contrast inverted Zeiss Microscope (Carl-Zeiss, Jena, Germany). Subsequently, the intercellular junctions were divided between the cell bodies to calculate the relationship between them (endothelial cell tube formation = cellular connections/cellular bodies). Non-stimulated cells were used as controls in the same conditions. Each experiment was performed in triplicate.

### Cellular viability

CPLMEC (5 × 10^5^ cells/plate) were plated on 35 mm culture plates and were grown for 4 days to obtain confluent cultures and treated with 1 μg/ml of DiSA or tDiSA for 24 h. Subsequently, hypoxia was induced for 48 h. Non-stimulated cells were used as controls in the same conditions. Cell counts were performed using the equipment Countess® Automated Cell Counter (Invitrogen) following the manufacturer’s instructions.

### Statistical analysis

The GraphPad Prism v.7 was used for all data analyses. Analyses were performed by ANOVA and corrected for repeated measurements when appropriate. If ANOVA revealed overall significant differences, individual means were evaluated *post-hoc* using Tukey’s test. All results were expressed as the mean ± SEM. In all experiments, a significant difference was defined as a *P*-value of < 0.01 for a confidence level of 99%.

## Results

### *Wolbachia* content of the antigenic extracts of *D. immitis* adult worms

**Fig. 1:**
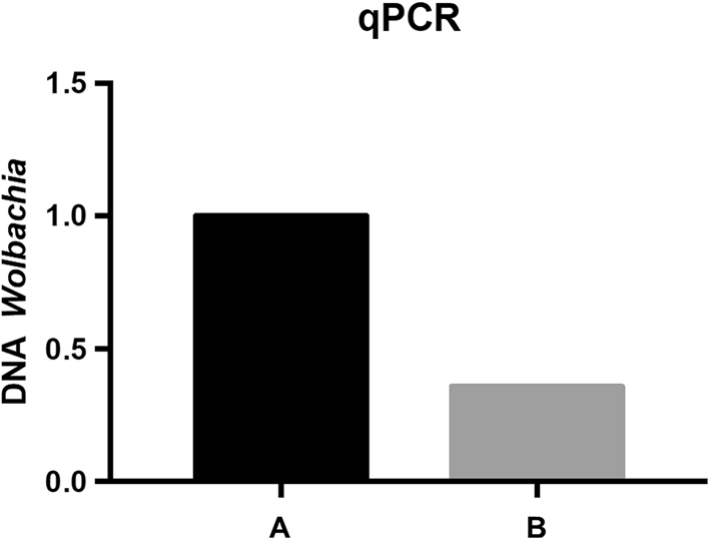
Quantification of *Wolbachia* DNA by qPCR in *Dirofilaria immitis* adult worms from an untreated dog (A) and a dog treated with doxycycline (B)

Quantification of the ftsZ gene expression of *Wolbachia* by qPCR in the antigenic extracts of adult worms of *D. immitis* from an untreated (DiSA) and a doxycycline treated dog with (tDiSA) is shown in Fig. 1. The amount of *Wolbachia* in worms from the treated dog was drastically reduced by approximately 60% when compared to worms from the untreated dog.

### Effect of DiSA and tDiSA extracts on cell viability

**Fig. 2:**
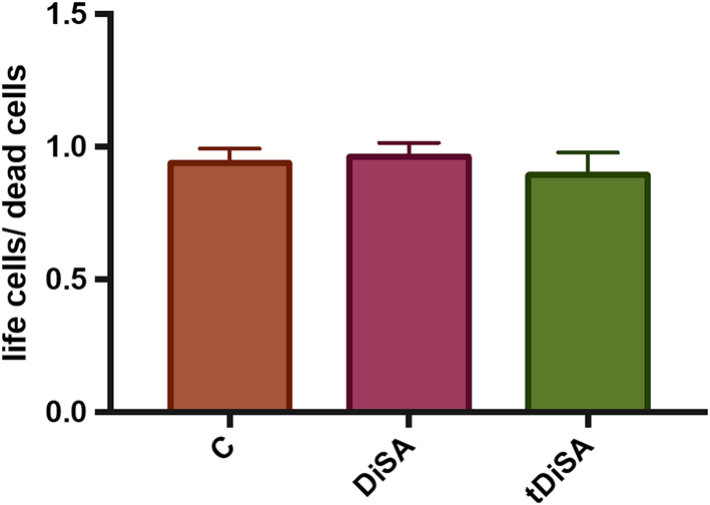
Effects of DiSA and tDiSA extracts on cell viability: non-stimulated cultures (C), cultures stimulated with DiSA and cultures stimulated with tDiSA. Results are expressed as the mean ± SD of 3 independent experiments

No differences were found in cell viability of cultures stimulated with DiSA or tDiSA compared to non-stimulated cell cultures (Fig. 2).

### Effect of DiSA and tDiSA extracts on angiogenic factors

A general view of the effects of DiSA and tDiSA on the expression of the different factors studied and pseudocapillary formation are shown in Table 1.

**Table 1:** Results obtained stimulating CPLMEC with the extract from *D. immitis* adult worms (DiSA and tDiSA)

	VEGF-A	sFlt	m-Endoglin	s-Endoglin	Pseudocapillary formation
DiSA	+	=	=	=	=
tDiSA	=	=	-	+	-

*Key*: +, upregulation; -, downregulation; =, no effect

#### VEGF-A

**Fig. 3:**
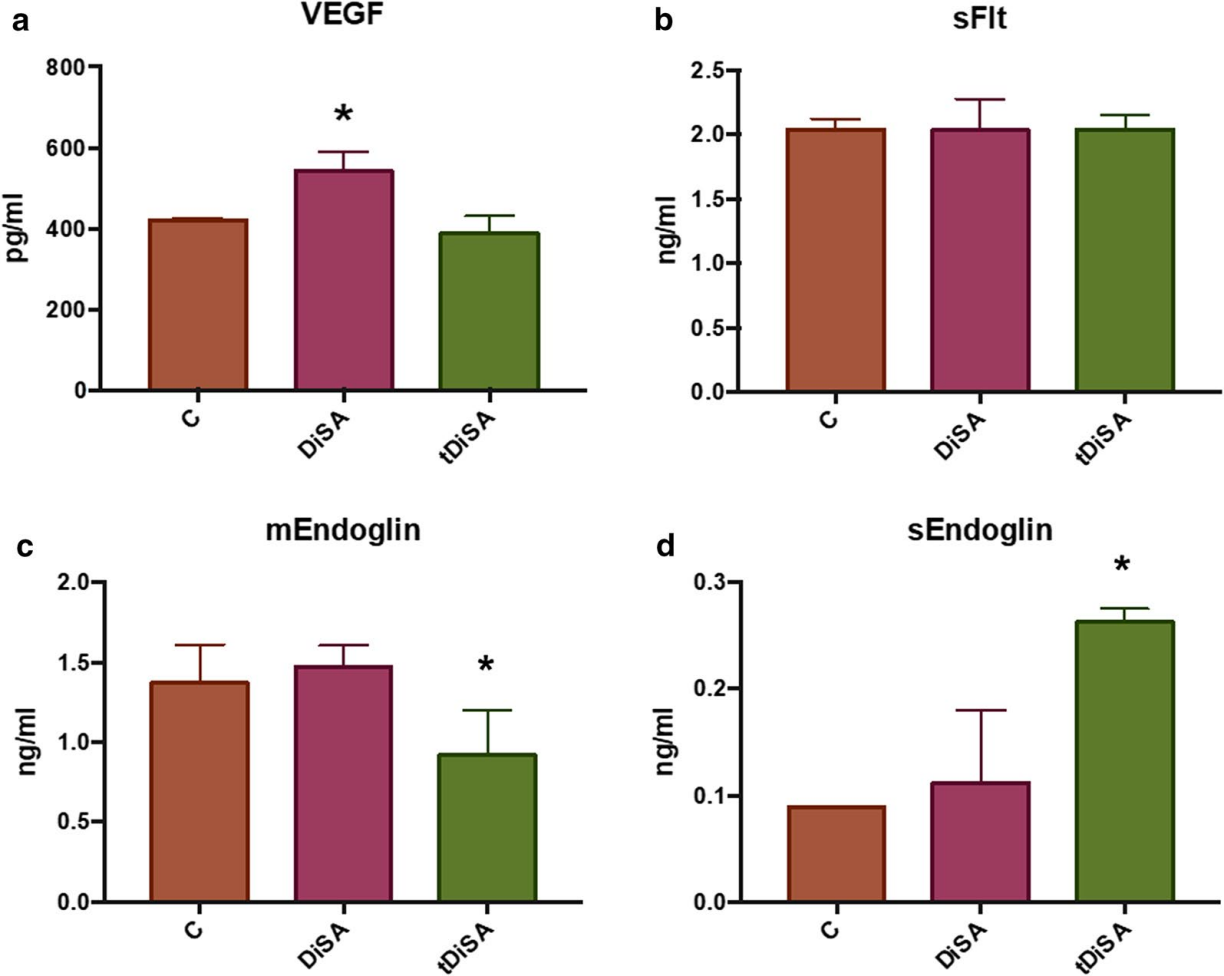
Expression of angiogenic factors VEGF-A (**a**), sFlt (**b**), mEndoglin (**c**) and sEndoglin (**d**), measured in supernatants of non-stimulated cultures (C), cultures stimulated with DiSA, or cultures stimulated with tDiSA. Results are expressed as the mean ± SD of 3 independent experiments. Significant differences in comparisons with the other groups are indicated by an asterisk (*)

The stimulation of cell cultures with DiSA increased the expression of VEGF-A when compared to non-stimulated cultures (Fig. 3a), while the stimulation with the tDiSA extract did not modify the expression of this angiogenic factor. There were significant differences between the content of VEGF-A present in the cultures stimulated with DiSA and the existing in both the non-stimulated control cultures and in cultures stimulated with tDiSA (*F*_(2, 10)_ = 19.64, *P* = 0.0003). No significant differences were observed between VEGF-A from unstimulated cultures and those stimulated with tDiSA.

#### sFlt

Neither of the two extracts significantly modified the expression of sFlt in the cultures of microvascular endothelial cells, compared to the non-treated controls (Fig. 3b).

#### mEndoglin

The expression of mEndoglin by the endothelial cells was modified by the tDiSA but not by the DiSA extract. Cultures stimulated with tDiSA showed a significant decrease in mEndoglin compared to non-stimulated controls and in cultures stimulated with DiSA (*F*_(2, 15)_ = 10.22, *P* = 0.0016) (Fig. 3c).

#### sEndoglin

Only tDiSA significantly modified the expression of sEndoglin (Fig. 3d). The cultures stimulated with tDiSA showed a significant increase in the expression of sEndoglin when compared to the non-stimulated cultures and cultures stimulated with DiSA (*F*_(2, 3)_ = 11.17, *P* = 0.00407) (Fig. 3d).

### Effect of DiSA and tDiSA extracts on pseudocapillary formation

**Fig. 4:**
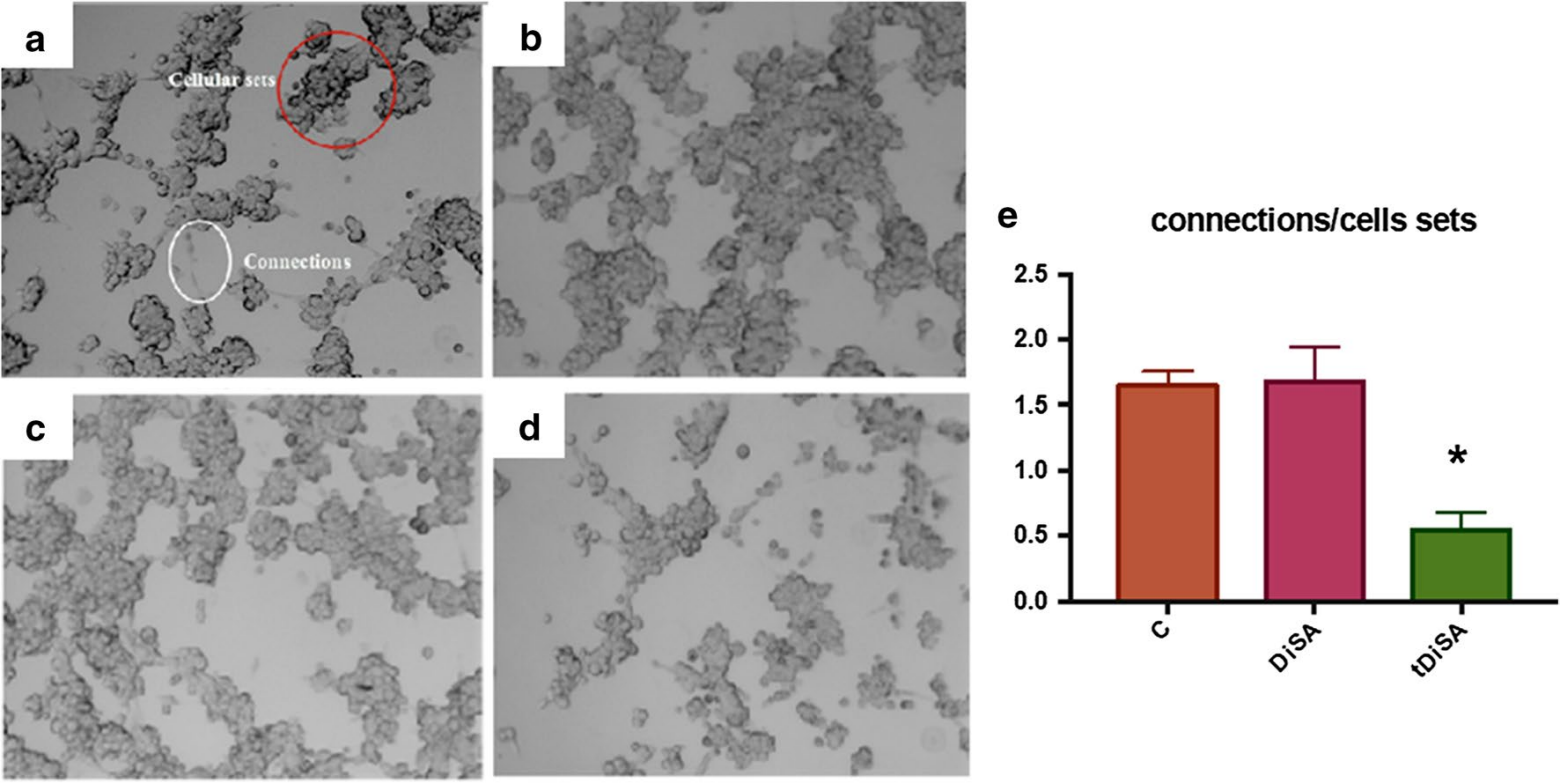
Endothelial cell tube formation assay on Matrigel plates. Representative image showing cellular connections and cellular sets evaluated in the experiment (**a**). Representative images from non-stimulated cultures (**b**), cultures stimulated with DiSA (**c**) and cultures stimulated with tDiSA (**d**). **e** Graphic representation of the cell connections/cell sets relationship in non-stimulated cultures (C), cultures stimulated with DiSA and cultures stimulated with tDiSA. Results are expressed as the mean ± SD of 3 independent experiments. Significant differences in comparisons with the other groups were indicated by an asterisk (*)

The capacity for pseudocapillary formation was evaluated by analyzing the cell junctions (connections) and the cell groups that emerged in stimulated and non-stimulated cultures (Fig. 4, Table 1). The formation of pseudocapillaries and the connections/joint relationship in cultures stimulated with DiSA were very similar to those present in the non-stimulated controls. However, the cultures stimulated with tDiSA were less organized and a drastic decrease in the formation of pseudocapillaries and cell junctions compared to non-stimulated cultures were observed. There was significant lower capacity of pseudocapillary formation in cultures stimulated with tDiSA and those observed in non-stimulated or stimulated with DiSA cultures (*F*_(2, 9)_ = 50.33, *P *< 0.0001 in both cases).

## Discussion

There are very few data on the ability of nematodes to induce angiogenesis. Some studies suggest that microfilariae and adult worms of lymphatic filariae induce lymphangiogenesis and *in vitro* remodeling of lymphatic channels [22, 23]. Furthermore, the study of subcutaneous dirofilariosis caused by *Dirofilaria repens* by ultrasound and Doppler techniques, demonstrated a clear remodeling of the blood vessels in the periphery of the dirofilariotic nodules [24].

Regarding heartworm disease, long-term inflammatory and obstructive alterations that occur in the pulmonary arteries lead to a reduction in the flow of blood, hypoxia, perivascular edema and pulmonary hypertension [9]. Some of the pathologic processes of heartworm disease are immune-mediated while some others are associated with the demonstrated capacity of the molecules of *D. immitis* and *Wolbachia* released from dead worms to modify the vascular environment of the worms [7, 25]. Nevertheless, to the authors’ knowledge, this is the first report that analyzes the influence of antigenic extracts of *D. immitis* adult worms, with different *Wolbachia* content, in the angiogenic process.

In a first step we demonstrated that the antigenic extract of worms from a dog treated with doxycycline (tDiSA) had much less *Wolbachia* than the extract of worms removed from an untreated dog (DiSA). Using an *in vitro* model of canine microvascular endothelial cells, we showed that these antigenic extracts of *D. immitis* are able to modify the expression of some important angiogenic factors as well as influence the formation of pseudocapillaries, without altering the cell viability. Furthermore, these effects were dependent on the amount of *Wolbachia* present in the antigenic extracts.

DiSA extract, with its content in *Wolbachia* intact, seems to have a proangiogenic effect, since it caused a significant increase in VEGF-A expression by endothelial cells. Furthermore, tDiSA, which presented a low amount of *Wolbachia*, did not modify the normal expression of VEGF-A. Studies on inflammatory mechanisms have shown an increase in the expression of VEGF in cultures of human aortic endothelial cells stimulated by *Wolbachia* surface protein (WSP) [12]. VEGF also increases in patients naturally infected by *Bartonella bacilliformis* [26]. In addition to its role in the transmigration of neutrophils and monocytes during the inflammation, VEGF is one of the main proangiogenic factors synthesized by endothelial cells [23]. VEGF performs its function by binding to the receptor tyrosine kinase VEGFR2 (Flk-1), which is responsible for proangiogenic signaling. In contrast, VEGFR1 (Flt-1) and its soluble form, sFlt-1, sequester the VEGF ligand by performing a negative regulation of the proangiogenic response [27].

In the present model, sFlt-1 was not modified by any of the used extracts, suggesting that it does not play any role, at least during the first 24 hours after the stimulus. Regarding endoglins, only the tDiSA produced effects on the expression of these factors, by significantly decreasing the expression of mEndoglin and increasing sEndoglin. mEndoglin, the form linked to the cell membrane, has a proangiogenic effect and its expression increases under physiological conditions during the vascularization of tissues, as well as in pathological conditions with presence of angiogenesis [28]. sEndoglin, originating from the proteolysis of the extracellular portion of mEndoglin, has shown anti-angiogenic activity in different processes [29], as well as its participation in endothelial dysfunction through its pro-inflammatory activity [21]. Therefore, our results suggest that a low amount of *Wolbachia* in the extracts may be related to an *in vitro* antiangiogenic effect through the downregulation of the proangiogenic mEndoglin and the upregulation of anti-angiogenic sEndoglin. Endothelial cells can form two-dimensional reticular structures when cultured in Matrigel plates [30]. These structures resemble the immature vessels formed during angiogenesis. In accordance with the results obtained regarding angiogenic factors, the trend of our cell cultures to form pseudocapillaries was negatively altered when stimulated with tDiSA, but no changes were detected in cultures stimulated with DiSA, probably as a consequence of incubation time, antigen concentration or other factors related to the limitations of the model.

## Conclusions

To the best of our knowledge, this study demonstrates for the first time the ability of the antigenic extracts of *D. immitis* to modify the expression of some angiogenic factors and the formation of pseudocapillaries *in vitro*. The capacity to stimulate these changes seems related to the amount of *Wolbachia* in these extracts. Given the complexity of the process, the variety of molecules and factors involved, more studies, e.g. on the influence of the antigenic extracts of *D. immitis* from dogs treated with an association of doxycycline and macrocyclic lactones on the process of pseudocapillary formation, as well as the correlation with the amount of *Wolbachia* in the angiogenic process, are needed to fully understand the role of *D. immitis* and *Wolbachia* in the angiogenic response. Moreover, our results seem to suggest that the therapeutic activity of doxycycline could be related not only to the anti-inflammatory but also to the anti-angiogenic effects associated with the decrease of *Wolbachia* bacteria residing in *D. immitis*.

## Data Availability

The datasets supporting the conclusions of this article are included within the article.

## Abbreviations

CPLMEC: canine primary lung microvascular endothelial cells
DiSA: extract of adult worms of *D. immitis* obtained from an untreated dog
mEndoglin: membrane endoglin
PCR: polymerase chain reaction
qPCR: real-time PCR
tDiSA: extract of adult worms of *D. immitis* obtained from a dog treated fifteen days with doxycycline
sEndoglin: soluble endoglin
sFlt: soluble fms-like tyrosine kinase 1
VEGF-A: vascular endothelial growth factor-A
